# Evaluation of the role of mitochondria in the non-targeted effects of ionizing radiation using cybrid cellular models

**DOI:** 10.1038/s41598-020-63011-w

**Published:** 2020-04-09

**Authors:** Silvana Miranda, Marcelo Correia, Anabela G. Dias, Ana Pestana, Paula Soares, Joana Nunes, Jorge Lima, Valdemar Máximo, Paula Boaventura

**Affiliations:** 1 i3S - Instituto de Investigação e Inovação em Saúde, Universidade do Porto, Rua Alfredo Allen, 208, 4200-135 Porto, Portugal; 2 Ipatimup - Institute of Molecular Pathology and Immunology of the University of Porto, Rua Júlio Amaral de Carvalho 45 4200-135, Porto, Portugal; 3 Radiotherapy Department, Portuguese Institute of Oncology of Porto (IPO Porto), Rua Dr. António Bernardino de Almeida, 4200-072 Porto, Portugal; 4 Medical Physics Department, Portuguese Institute of Oncology of Porto (IPO Porto), Rua Dr. António Bernardino de Almeida, 4200-072 Porto, Portugal; 5 Medical Physics, Radiobiology and Radiation Protection Group. Research Center, Portuguese Institute of Oncology of Porto (IPO Porto), Rua Dr. António Bernardino de Almeida, 4200-072 Porto, Portugal; 6 Faculty of Medicine, University of Porto, 4200 - 319 Porto, Portugal; 7 Department of Pathology, Faculty of Medicine, University of Porto, 4200 - 319 Porto, Portugal; 8 Department of Pathology, Leiden University Medical Center, Leiden, The Netherlands

**Keywords:** Cell biology, Biological models

## Abstract

Radiobiology is moving towards a better understanding of the intercellular signaling that occurs upon radiation and how its effects relate to the dose applied. The mitochondrial role in orchestrating this biological response needs to be further explored. Cybrids (cytoplasmic hybrids) are useful cell models for studying the involvement of mitochondria in cellular processes. In the present study we used cybrid cell lines to investigate the role of mitochondria in the response to radiation exposure. Cybrid cell lines, derived from the osteosarcoma human cell line 143B, harboring, either wild-type mitochondrial DNA (Cy143Bwt), cells with mitochondria with mutated DNA that causes mitochondrial dysfunction (Cy143Bmut), as well as cells without mitochondrial DNA (mtDNA) (143B-Rho0), were irradiated with 0.2 Gy and 2.0 Gy. Evaluation of the non-targeted (or bystander) effects in non-irradiated cells were assessed by using conditioned media from the irradiated cells. DNA double stranded breaks were assessed with the γH2AX assay. Both directly irradiated cells and cells treated with the conditioned media, showed increased DNA damage. The effect of the irradiated cells media was different according to the cell line it derived from: from Cy143Bwt cells irradiated with 0.2 Gy (low dose) and from Cy143Bmut irradiated with 2.0 Gy (high dose) induced highest DNA damage. Notably, media obtained from cells without mtDNA, the143B-Rho0 cell line, produced no effect in DNA damage. These results point to a possible role of mitochondria in the radiation-induced non-targeted effects. Furthermore, it indicates that cybrid models are valuable tools for radiobiological studies.

## Introduction

Ionizing radiation (IR) is used in cancer therapy due to its ability to control tumor growth by inducing DNA damage. In the last decades, it has been demonstrated that the effects occurring in bystander non-irradiated cells which receive signals from irradiated counterparts, mimic those happening in directly irradiated cells^1–3^. This phenomenon is commonly denominated as the non-targeted effects (NTE) or bystander effects of irradiation^4^. This intercellular communication can occur *via* intercellular gap junctions – with a dependence on the connexins expressed by the irradiated cells and their ability to communicate this stress stimulus (irradiation) to neighbor cells^5^; and/or *via* the release of factors directly or *via* exosomes to the extracellular media that can reach cells further away from the releasing cells^6–9^.

Nagazawa and Little, who described the occurrence of chromosomal aberrations in the progeny of cells that were irradiated with alpha particles, were among the first bringing the attention to the effects of DNA damage that are not a direct consequence of IR exposure^10^. The chromosomal aberrations, observed in the form of sister chromatid exchanges, resulted from very low levels of exposure, suggesting that only a small fraction of the initial cells were irradiated, and lasted for several generations after irradiation^10^.

A possible mechanism related to these effects would be intercellular signaling mediated by factors released from irradiated cells, which could trigger a response in neighboring cells^11^. However, the nature of the released signals is still unclear. Several factors have been proposed: typical inflammatory cytokines such as interleukin 6 (IL6) or other molecules involved in inflammation, like pro-apoptotic cytokine Fas-L, could be responsible for the alterations observed in non-irradiated cells^12^. Nitric oxide (NO) also constitutes a possible vehicle through which irradiated cells activate response processes in adjacent non-irradiated cells^13^. It was shown that a NO scavenger – 2-(4-carboxyphenyl)-4,4,5,5-tetramethylimidazoline-1-oxyl-3-oxide (c-PTIO) – is able to decrease micronuclei formation in neighboring cells after IR^14^. NTE in the form of mutational load were lower when Bay 11–7082, a pharmacological inhibitor of nuclear factor-κB (NF-κB) activation, was used, indicating another candidate for bystander signaling mechanism^15,16^. Reactive oxygen species (ROS), important signal molecules and key players in cellular homeostasis^17^, are another possibility for the signaling transduction^7^ as well as oxidized DNA fragments^18^ and cell free chromatin, shown to induce a response in non-irradiated cells *via* the NF-E2 related factor-2 (NRF2)^19^. There is also evidence for a role of purinergic mechanisms activating DNA damage receptors^20^. Another possibility lies in the release of microRNAs (such as miR-21) by the irradiated cells which will increase DNA damage in bystander cells^21^. In fact, miRNAs are described as key players in the gene regulation in response to cellular irradiation^8^. Exosomes, a form of extracellular vesicles (EVs) that are released by cells under various conditions as a form of extracellular communication, are cited in various contexts as carriers of some of the aforementioned molecules^22–24^. Table 1 lists proposed candidates of bystander cell signals. Recent work has shined light into a particular type of cellular communication, one that occurs *via* electromagnetic radiation in the ultra violet (UV) light spectrum^25^. These *biophotons* are emitted by biological material and have been described to occur as a response to stress. In the context of radiation and NTE, they have been implicated as a possible mechanism by which cells alert others about radiation-induced changes^26^. Le *et al*. verified that cells exposed to IR emit *biophotons* which incite the release of exosomes on the bystander cells^24^.

**Table 1:** List of signals that have been proposed as NTE potential mediators.

Proposed signal mechanism	Brief description	Author; year	
Nitric Oxide	Due to NO lipophilic nature and stability, it constitutes a possible vehicle with which irradiated cells activate response processes in adjacent non-irradiated cells; increase in micronuclei formation after IR was abrogated when a NO specific scavenger was used.	^13,14^	
Nuclear Factor kappa B	Upon inhibition of NF-κB, a decreased frequency of mutations was observed in the cells studied.	^16^	
Reactive oxygen species	ROS scavengers reduced the frequency of DNA double strand breaks (DSB) in cells subjected to media collected from irradiated cells.	^7^	
Purinergic	Upon release from the cells act as intercellular signaling molecules in what is known as purinergic signaling, shown to be important in the response to IR- Their work also shown that ATE released from irradiated cells activate receptors in non-irradiated cells which are involved in DNA damage and repair response.	^20,46^	
Biophotons	Radiation in the ultra violet (UV) light spectrum. These *biophotons* are emited by biological material as a response to stress. In the context of radiation and NTE, they have been implicated as a possible mechanism by which cells *alert* others about radiation-induced changes.	^24,26,30^	
Oxidized extracellular DNA	Oxidized DNA fragments stimulate an increase in ROS production which leads to an adaptive response *via* nuclear translocation of NF-E2 related factor-2 (NRF2) and consequent antioxidant enzymes activation in non-irradiated cells.	^18,47^	
Cell free Chromatin	Cell free chromatin that is released from dying cells is able to initiate DNA damage and inflammation in the neighbor cells.	^19,48^	
Extracellular vesicles carrying:	**MicroRNAs**	Key players in the gene regulation in response to cellular irradiation.	^8,21^
	**Mitochondrial DNA**	EVs from irradiated cells that lack mitochondrial DNA (mtDNA) are not able to increase the levels of DNA damage in bystander (non-irradiated) cells.	^9^

It seems plausible that the radiation-related signaling is part of an integrated complex response to stress, which is used to alert and improve the adaptation of the cellular population, in order to maintain cellular homeostasis. Mitochondrial function appears to be linked to the efficacy of these mechanisms at several levels. In fact, mitochondria are not only responsible for ATP synthesis, they are also essential for other cellular tasks, such as fatty acid metabolism^27^, Ca^2+^ homeostasis^28^, apoptotic cascades^29^, among others. Moreover, in a recent work, Ariyoshi *et al*. proposed another important role for mitochondria in NTE. They observed that irradiated cells release mitochondrial (mtDNA) into exosome-like vesicles that act as signals to the bystander cells. Accordingly, irradiated cells lacking mtDNA were not able to increase DNA damage levels in bystander (non-irradiated) cells, which reinforces the assumption of mtDNA as a bystander signal upon irradiation^9^. Additionally, the function of the mitochondrial complex I was shown to be disrupted in bystander cells as a response to biophotons-mediated NTE^30^, which suggests that mitochondria can not only be players in NTE mediation, but also susceptible to NTE signals.

Cytoplasmic hybrid, or cybrid, cell lines were developed as models for studying mtDNA influence in a myriad of cellular processes^31^. Cybrids share the same nuclear DNA background but differ in their mitochondrial DNA content, allowing a proper distinction of the effects caused from specific mtDNA alterations^31^. Cybrids are important tools for studying diseases arising from mitochondrial dysfunction, as reviewed by van Gisbergen *et al*.^32^, but they are also interesting *in vitro* models to study the role of mitochondria in cellular processes^33^. In line with this, we hypothesize that cybrids can serve as good models to understand the role of mitochondria in the signalling mechanisms occurring upon IR.

In a previous work from our group, osteosarcoma cybrids carrying an adenine to thymine transition in the position 3243 in mtDNA were studied. This gene encodes for the tRNA for leucine and this alteration was shown to cause the cells to exhibit altered metabolism (lower oxygen consumption, and higher glucose consumption and lactate production), increased ROS levels, and increased motility and migration capacities^34^. In this study we used these cell models for studying the effects of IR as an anti-cancer therapy, as they have altered mitochondrial function^34^.

We hypothesized that altered mitochondrial function changes response to direct irradiation and could also change the bystander effect through changes in irradiated cells conditioned media composition.

## Materials and Methods

### Cell culture

The three cell lines used in this study were Cy143Bwt, Cy143Bmut and 143B-Rho0. The143B-Rho0 cells (devoided of mtDNA) were obtained from the 143B osteosarcoma-derived cell line after transient expression of UL12.5 Herpes Simplex protein, leading to mtDNA degradation^35^. The Cy143Bwt cybrid cell line was obtained by fusing 143B-Rho0 cells with human XTC.UC1 enucleated cells and subsequent isolation of single cell clones harbouring WT mtDNA. The Cy143Bmut was obtained by fusing 143B-Rho0 cells with human platelets from a patient with encephalopathy disease carrying the A3243T mtDNA mutation in the mitochondrial tRNA^lLeu(UUR)^ gene. All the three cell lines share the same nuclear background and only differ in their mtDNA content.

143B-Rho0 were established at Dr Keshav Singh Lab [University of Alabama at Birmingham (UAB), Alabama (AL), United States of America (USA)]. The other two cybrid cell lines used were established by our group^34^.

143B-Rho0 cells are pyrimidine and pyruvate auxotrophs and therefore need to be cultured in medium supplemented with uridine and pyruvate. For a matter of consistency, all cell lines were cultured in Dulbecco’s Modified Eagle’s Medium (DMEM) high-glucose (Capricorn, Ebsdorfergrund, Germany) supplemented with 10% (v/v) inactivated and filtered Fetal Bovine Serum (FBS), 1% (v/v) Penicillin/Streptomycin (PenStrep, Biowest, Nuaillé, France), 0.5% fungizone (Biowest), 50 μg/ml uridine (Sigma, MO, USA) and 100 mM Sodium Pyruvate (Sigma). Cells were routinely kept in culture at 37 °C, 5% carbon dioxide (CO_2_), in a humidified incubator.

### Cell line characterization

For cellular growth curve determination, 4.0 × 10^4^ cells were plated in 6 well plates. Every 24 hours, cells were collected and counted in a Z2 Coulter Particle Counter (Beckman Coulter, Brea, CA). Briefly, cells were washed once with phosphate buffered saline (PBS) 1x and detached using Gibco TrypLE Express (ThermoFisher Scientific). Cells were then re-suspended in culture medium and collected. After a 1:200 dilution in COULTER ISOTON II Diluent solution, cell number was determined in a Z2 Coulter particle counter (Beckman Coulter, Brea, CA). In order to establish a basal cell growth curve, at least three replicates were conducted. The same protocol was applied after IR exposure. The experiments were repeated three times.

### DNA extraction, PCR and sequencing

DNA extraction was performed using GRS Genomic DNA Kit – Blood & Cultured Cells according to the manufacturer’s instructions (Grisp Research Solutions, Portugal) for DNA isolation from cell pellets. Nucleic acid concentrations were determined using the NanoDrop ND-1000 Spectrophometer (Nanodrop Technologies, Inc., DE, USA). mtDNA tRNA^Leu(UUR)^ mutation was assessed by PCR followed by direct sequencing. The primers used for amplification covered the region of the A3243T mutation of the tRNA^Leu(UUR)^ gene, forward (FW) primer (5′-ACACCCACCCAAGAACAGGGTTT- 3′) and reverse (RV) primer (5′-GTAGAATGATGGCTAGGGTACT-3′) (Invitrogen/ThermoFisher, MA, USA). PCR reactions were performed in total reaction volumes of 25 μl using ~100 ng of DNA, 0.1 μM of each FW and RV primers, 1x PCR Buffer (5x GoTaq Flexi Buffer, Promega), 1.5 mM of magnesium chloride solution (Promega), 40 mM deoxyribonucleotide triphosphate (dNTP) mix (Bioron GmbH), and 0.5 U of GoTAq DNA polymerase (Promega). PCR reactions were performed on BIO RAD MyCycle thermal cycler (BIO RAD). PCR conditions were: 1 cycle of 5 minutes at 94 °C for initial denaturation, followed by 35 cycles of 30 seconds at 94 °C for denaturation, 30 seconds at 58 °C for annealing and an extension step of 30 seconds at 72 °C; the final extension consisted of 1 cycle of 5 minutes at 72 °C. PCR products were purified using 1 U/μl exonuclease I and 0.05 U/μl shrimp alkaline phosphatase (Fermentas) at 37 °C for 20 minutes, followed by heat inactivation at 80 °C for 15 minutes.

The sequencing reaction consisted of 0.5 μl of BigDye Terminator (Perking-Elmer, CA, USA), 3.4 μl of sequencing buffer (Perking-Elmer), 0.3 μl of primer (FW and RV for tRNA^Leu(UUR)^ analysis), 2 μl of purified PCR product and Dnase/Rnase-free distilled water (GIBCO) in a final volume of 10 μl. The sequencing reaction was performed in a BIO RAD MyCycler thermal cycle (BIO RAD) with the following conditions: an initial denaturation step of 10 seconds at 94 °C, followed by 35 cycles of 10 seconds at 94 °C, 30 seconds at 56 °C for the annealing, and elongation of 2 minutes at 60 °C; the final elongation was performed for 10 minutes at 60 °C. Before loading on the ABI Prism 3130XL Automatic Sequencer (Perking-Elmer), the products were purified by precipitation using Sephadex columns (Sephadex G-50 Fine, GE Healthcare Bioscience AB, Uppsala, Sweden) according to the manufacturer’s instructions. 15 μl of formamide (Applied Biosystems, CT, USA) were added to each pellet in order to maintain the DNA in a single stranded conformation.

### Cell irradiation and transportation

Cells were platted and left to grow for 24 h before irradiation. The irradiation protocol comprises two single doses: low dose 0.2 Gy; and high dose 2.0 Gy. Since the irradiation place was different from the place where the subsequent studies were done, cells had to be transported from Instituto de Investigação e Inovação em Saúde (i3S) to Instituto Português de Oncologia (IPO) Porto, where they were irradiated. Transport was done inside a container that kept optimal culture conditions. A stereofoam box was used for the transportation of cells, filled with the heated thermo-accumulators, and transported by foot through a 1.1 kilometers (Km) distance, 13 minutes (min) each way. For every irradiation, a sham control was included in the transport.

### Media transfer

A total of 2.5 × 10^5^ cells were plated in T25 cell culture flasks for media transfer experiments. Twenty-four hours after platting, cells were irradiated according to the conditions referred above and kept in the incubator for 60 minutes in normal cell-culture conditions: 37 °C, 5% CO_2_ and humidified atmosphere. The media were then collected and filtered with 0.22 μM polyethersulfone (PES) filters (Frilabo). Cells receiving the irradiated cells conditioned media (ICCM) were plated at the same time as the cells that were directly irradiated, kept in the incubator, and their media was replaced by the filtered ICCM.

### γH2AX evaluation

Cells were plated in 6 mm coverslips for incubation with the γH2AX antibody. The phosphorylated form of the γH2AX histone was evaluated in directly irradiated cells and in cells exposed to ICCM. One hour after irradiation, media was removed and cells were fixed for 30 min at room temperature with a 4% paraformaldehyde (PFA) solution. After three washes with PBS 1×, samples were kept at 4 ^o^C. Cells treated with ICCM were submitted to the same protocol. The primary antibody was anti-human Phospho-Histone γH2AX (S139) [affinity-purified polyclonal rabbit immunoglobulin G (IgG) from R&D Systems] and the secondary antibody was goat anti-rabbit IgG H&L with fluorescent dye (Alexa Fluor 594, AbCam).

The coverslips were removed and washed twice in PBS 1x before adding blocking buffer solution (1% Bovine Serum Albumin – BSA, 0.01% Triton X-100) for 30 min. After a new wash with PBS 1×, lamellae were incubated with the primary antibody (1:250 in blocking buffer) for 60 min, washed again with PBS and incubated with the secondary antibody (1:200 in blocking buffer) for 60 min. After the final washing with PBS, the lamellae were mounted on microscope laminae with Vectashield combined with 4’,6-diamidino-2-phenylindole (DAPI) (Vector Laboratories), sealed, and stored at −20 ^o^C for posterior analysis. Cells were visualized in a Zeiss Axio Imager Z1 fluorescent microscope. Images were collected, 10 cells for each condition, with 630 amplification and analyzed with ImageJ Version 1.51, to assess the number of γH2AX *foci*. Both direct IR and ICCM experiments for γH2AX evaluation were repeated three times.

### Cell irradiation with EBT3T radiochromic film dose control

24 hours after platting, cells were irradiated with a 6MV photon beam in a linear accelerator – Novalis Tx from Varian Medical Systems. The cell culture flasks or plates were irradiated with two different doses, 0.2–2.0 Gy, depending on the experiment. The flasks were centered with the irradiation field, and exposed at a source-surface distance (SSD – cells surface) of 100 cm, for a field size of 16×16 cm, with a dose rate of 0.4 Gy/min.

The doses were calculated considering the output factor (OF) for the field size used, the percentage depth dose (PDD) at depth of 2 cm for the field size of 16×16 cm and the day dose measured in the accelerator, to eliminate the accelerator dose variation.

In order to ensure a full backscatter condition in the experiment, a backscatter correction factor of 1.5% in the dose was used for the 6MV megavoltage photon beam, according to Yida Hu *et al*.^36^.

The monitor units (MU) used to administer the dose were calculated as follows:

$${\rm{MU}}=[({\rm{Dose}}/{{\rm{PDD}}}_{16\times 16@2{\rm{cm}}})/{{\rm{OF}}}_{16\times 16{\rm{cm}}}]\times {{\rm{Dose}}}_{({\rm{day}}{\rm{dose}})}$$

### Dose verification

In order to validate the cells irradiation, a dose control was performed using EBT3 radiochromic film. The film was placed below the cell culture flask, for each dose used. After irradiation, the films were kept from solar light for 24 hours until processing in EPSON Expression 10000XL scanner (conversion from film to digital image).

The results were obtained relating the film optical density (OD – film darkening) with dose, using for that a DoseLab software (Mobius Medical Systems, LP).

### Statistical analysis

Whenever adequate, the results were presented as mean ± standard deviation or mean ± standard error. Cell line experiments were analyzed with Two-Way ANOVA followed by the Bonferroni post-test correction available in Graph Pad Prism software version 5. A *p* value <0.05 was considered as statistically significant.

## Results

**Figure 1:**
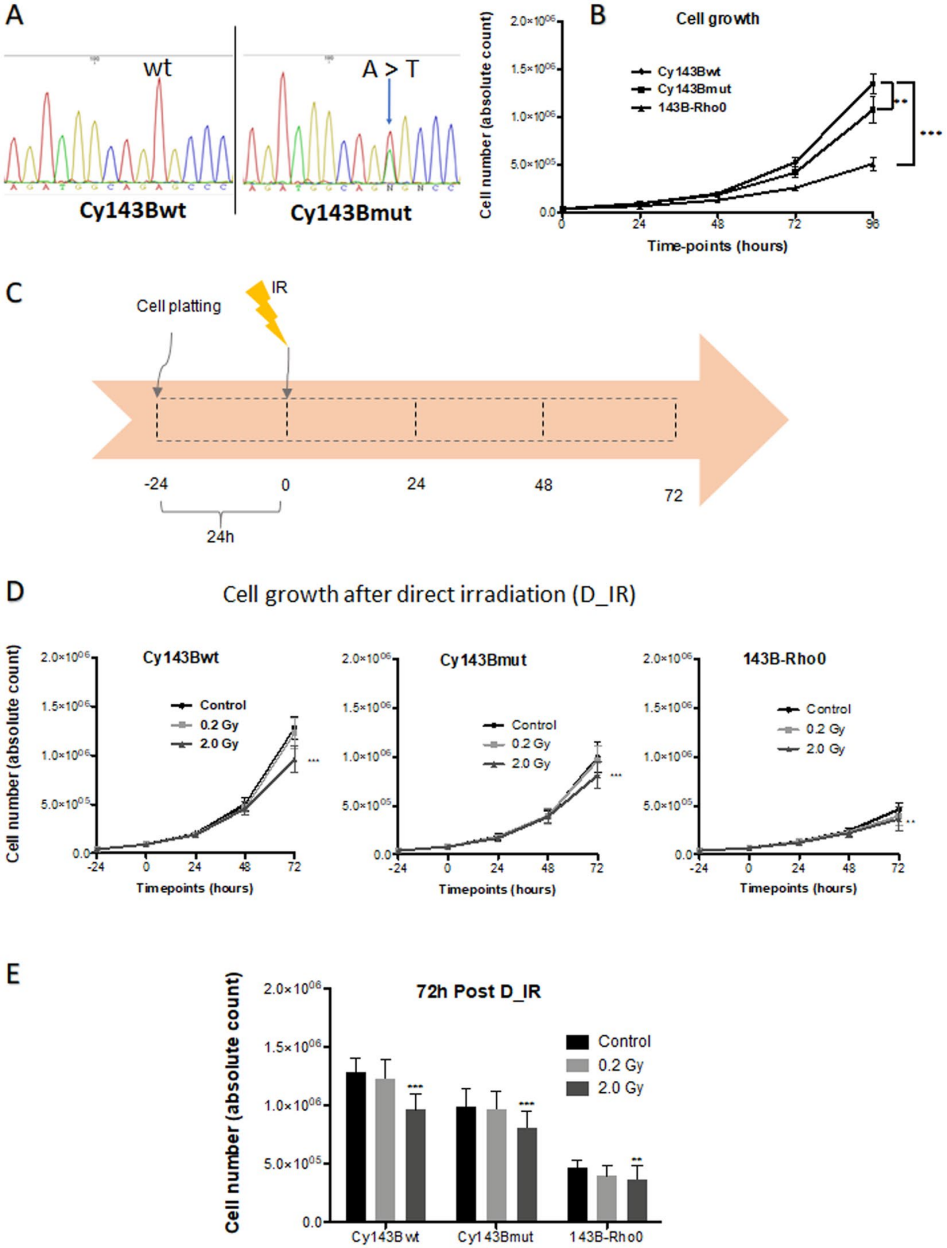
Cellular characterization and cell growth after direct irradiation. (**A**) DNA sequencing results for Cy143Bwt, showing the nucleotide Adenine (**A**) in the 3243 position; Cy143Bmut showing an Adenine to Thymine (T) transition at the 3243 position, with approximately 60% of heteroplasmy (blue arrow). (**B**) Absolute cell number counted for each cell line – Bars represent the standard error. Control Cy143Bwt (circle), Cy143Bmut (square), 143B-Rho0 (triangles). Differences to control statistically significant, with ** for p values <0,01 and *** for p values <0.001. Data subjected to two-way ANOVA and posterior Bonferroni test. (**C**) Schematic representation of the protocol used for evaluation of the direct irradiation (D_IR) effects. (**D**) Absolute cell number counted at each timepoint for the three cell lines after irradiation. Differences to control statistically significant, with ** for p values <0,01 and *** for p values <0.001. Data subjected to two-way ANOVA and posterior Bonferroni test. E – Bar graphic representation of absolute cell number of non-irradiated cells (black bars); irradiated cells (light grey  for 0.2 Gy and dark grey for 2.0 Gy) at 72 h after IR. Bars correspond to mean ± standard deviation. Differences to control statistically significant, with ** for p values <0,01 and *** for p values <0.001. Data subjected to two-way ANOVA and posterior Bonferroni test.

In line with previous data^34^, sequencing analysis confirmed the presence of the A3243T tRNA^Leu(UUR)^ mutation in Cy143Bmut cells, with approximately 40% of heteroplasmy, while the Cy143Bwt cells did not show the mutation (Fig. 1A).

As cellular growth rate can influence the response to radiation, we plotted the basal cellular growth for each cell line, to understand how the differences in mitochondrial status could influence their division. We observed that all cell lines grew exponentially, although at different rates. The Cy143Bwt was the one with higher increase in total number of cells, followed by Cy143Bmut, whereas 143B-Rho0 cell line showed the lowest growth rate (Fig. 1B). To understand the susceptibility of each cell line to irradiation, we irradiated all cell lines with 0.2 Gy and 2.0 Gy (Fig. 1C) and evaluated the cellular growth of each cell line (Fig. 1D,E). After IR, all cell lines exposed to 0.2 Gy showed a tendency to grow less than control cells. Irradiation with 2.0 Gy significantly reduced the cell growth of all cell lines compared with control cells (Fig. 1D,E).

**Figure 2:**
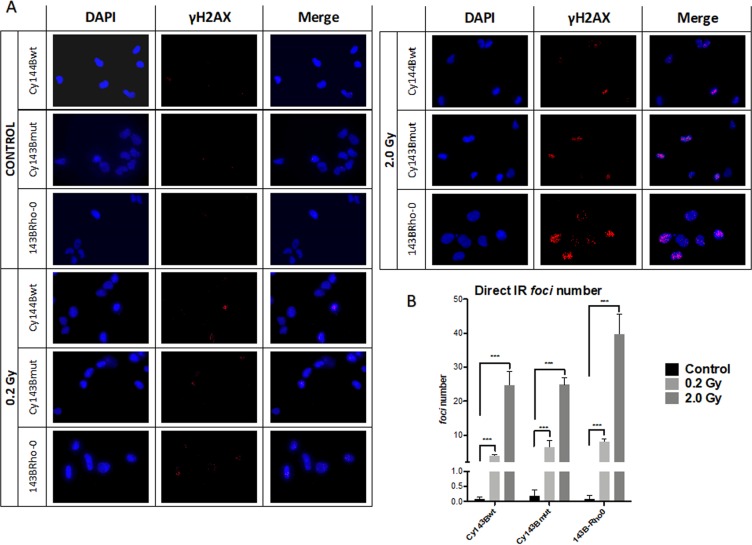
Evaluation and quantification of DNA damage after direct IR by γH2AX immunofluorescence. (**A**) Images obtained in the fluorescence microscope, showing localization of γH2AX foci (red fluorescence) in the nucleus (blue fluorescence, DAPI). Images were taken with the 63x objective. (**B**) Quantification of DNA DSB. The number of γH2AX foci was counted and a comparison between non-irradiated cells (black bars), and irradiated cells (light grey for 0.2 Gy and dark grey for 2.0 Gy). Bars correspond to mean ± standard deviation. Data subjected to two-way ANOVA and posterior Bonferroni test; p values <0,001 (***) (irradiated vs control).

The most important form of damage from IR are DNA DSBs. Basal levels and direct IR damage of nuclear DNA was evaluated using the γH2AX assay. *Foci* number were counted for controls and at different IR doses (0.2 Gy and 2.0 Gy) (Fig. 2A). After IR, all cell lines showed a statistically significant increase in the number of γH2AX *foci* over to their respective non-irradiated controls (*p* value <0.001), an effect that was more evident when using the 2.0 Gy IR dose (Fig. 2B). 143B-Rho0 cell line had the highest increase in γH2AX *foci* after IR with 2.0 Gy.

**Figure 3:**
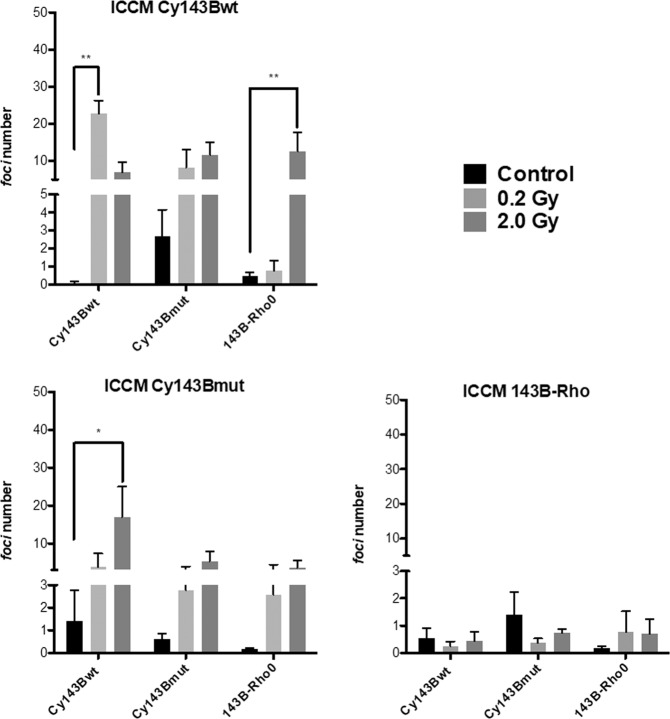
Number of γH2AX foci after treatment with ICCM. After 1 hour with ICCM, cells were stained with γH2AX antibody and the number of DSBs counted. Non-irradiated cells media (black bars); irradiated cells ICCM (light grey for 0.2 Gy and dark grey for 2.0 Gy). Bars correspond to mean ± standard deviation. Data subjected to two-way ANOVA and posterior Bonferroni test, p value <0,01 (**) and <0,05 (*).

Our main aim was to study the role of mtDNA status on the NTE (or bystander effects). To understand if the irradiated cybrid cells could induce damage and trigger cell death in bystander cells, we also investigated the induction of nuclear DNA DSBs in response to ICCM. In these assays, the control was the group of cells receiving media from non-irradiated cells which were submitted to the same conditions as the irradiated cells. ICCM from Cy143Bwt induced a significant increase in the number of γH2AX *foci* in Cy143Bwt and 143B-Rho0, when irradiated with 0.2 Gy and 2.0 Gy, respectively. No significant increase in γH2AX *foci* was observed in Cy143Bmut incubated with ICCM from Cy143Bwt cells, either irradiated with 0.2 Gy or with 2.0 Gy. ICCM obtained from Cy143Bmut cells irradiated with 2.0 Gy induced a statistically significant increase of γH2AX *foci* in Cy143Bwt cells, comparing with those induced with ICMM obtained after irradiation with 0.2 Gy, while Cy143Bmut and 143B-Rho0 cells did not shown increase of γH2AX *foci*, compared with controls. ICCM collected from 143B-Rho0 cells irradiated with 0.2 Gy or 2.0 Gy was not able to increase DSBs in any cell line (Fig. 3).

**Figure 4:**
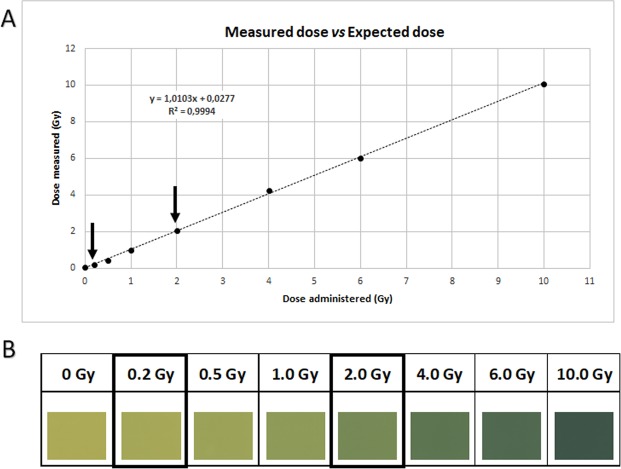
Verification of the irradiation doses. (**A**) Comparison of the dose expected and measured for irradiation. The relation between the programmed dose and the dose measured with EBT3 films is very close to 1, confirming the accuracy of the irradiation dose used in the experiments (dark arrows). (**B**) Scans of the EBT3 radiochromic film irradiated. The graph in A was plotted using the color reference for each dose.

Assuring the conditions of irradiation, namely the dose, were the same for each experiment, a rigorous protocol for verification was implemented. Dose measurements were performed using EBT3 films. These were irradiated with different doses within the range of 0–10 Gy. The results were obtained relating the film OD with the dose, using DoseLab software. We can observe the film darkening with increased dose. The plot relating the dose programmed and the dose read through this method showed a close correlation between the dose programed and the dose delivered (r2 = 0,99938) (Fig. 4A). The exposure of the films to the IR was compared with the calibration curve previously obtained (Fig. 4B).

## Discussion

NTE are not yet well understood and the nature of the signals transmitted after IR inducing NTEs in cells remains to be elucidated. Mitochondria are essential organelles that participate in the regulation of a myriad of cellular functions. Of interest to this study, is the fact that mitochondria regulate the response to cell insults, namely radiation damage^37^. In the NTE context, it was suggested that cells without mitochondria or with impaired mitochondrial function do not produce, upon irradiation, factors that induce NTE in non-irradiated cells^38^. In the present work we addressed the influence of mitochondrial dysfunction on NTE signaling, using cybrid cell lines as models, thus ensuring that the observed variations in response to IR are dependent of mitochondria and independent of nuclear background. In our study we included a cybrid cell line harbouring the A3243T mtDNA mutation in the mitochondrial tRNA^Leu(UUR)^gene (Cy143Bmut), a cybrid cell line harbouring WT mtDNA (Cy143Bwt) and the 143B-Rho0 which lacks mtDNA and gave rise to the other two cybrid cell lines. The use of cybrid cells allows the study of IR and its bystander effects in cells sharing the same nuclear background^31,39^. Under basal conditions, the Cy143Bwt cells display the higher cellular growth, while the 143B-Rho0 cells display the lower cellular growth rate. This is in line with other reports^34,40^ and demonstrates that mtDNA alterations, and consequent disruption of the mitochondrial oxidative phosphorylation, have a negative impact in cellular growth.

The correlation between mitochondrial dysfunction and DNA damage is still poorly understood in the radiation context. Since the cybrid cells show different cellular growth rates, we wondered if the response to direct exposure to IR could also be different. Direct irradiation had a negative impact in cell growth in all cell lines. The presence of mtDNA mutations has been associated with higher sensitivity to IR^41^, nevertheless, concerning cybrid cell line growth, our results seem to indicate that all cybrid cell lines were similarly sensitive to IR. Also, we did not observe any differences in DSBs formation, when comparing between mtDNA mutated cybrid and the *wt* cybrid, while 143B-Rho0 cells had a higher degree of DSBs formation, comparing to the former cell lines. The radiosensitivity of cells depleted of mtDNA is a subject of controversy, with reports suggesting that these cells would be more sensitive and others suggesting the opposite^40,42,43^. It has already been shown that after irradiation, the oxidative energy production is increased in order for the cells to have more ATP for a correct DNA repair^44^, which could explain why, in our work, 143B-Rho0 cells showed more DSBs after IR, although this needs to be further investigated. The oxidative energy production in the 143B-Rho0 is presumably strongly restricted and thus another possible cause for the increased amount of DNA damage.

Next, to explore NTE of IR, we used ICCM, one of the most employed methods to mimic NTE signalling and response. Considering the fact that cells may respond to ICCM in a similar manner as if they had been irradiated directly^1–3^, we assessed DNA damage in bystander cells. ICCM from Cy143Bwt and Cy143Bmut irradiated cell lines increased DNA damage; however, different effects were observed in response to ICCM from the three cell lines submitted to the same irradiation dose. This could mean that mitochondrial status may affect the ICCM composition and NTE. Considering the effect of Cy143Bwt ICCM in the three cell lines, we hypothesized that if the irradiated cells have *wt* mtDNA, low doses trigger IR-related signalling to alert neighbour cells and initiate adaptive responses. We postulate that this signalling mechanism might be mitochondria-dependent because we detected a lower amount of DNA damage in response to ICCM from impaired mitochondria cells (Cy143Bmut and 143B-Rho0), subjected to the same low doses. Nevertheless, Cy143Bmut could signal after high dose irradiation, suggesting that their alert mechanisms could be less effective or other stress response mechanism may be activated. ICCM from the 143B-Rh0 did not increase DNA damage, which sustains our hypothesis that NTE may be, at least in part, mediated by functional mitochondria. The absence of mtDNA renders irradiated cells unable to produce/activate any elements capable of inducing DNA damage. Our assumption that the ability of cells to produce or to release a signal after IR may depend on mitochondria is in line with Tartier *et al*. who showed that cells lacking mtDNA, when exposed to radiation, are not able to signal this insult to other cells. However, these cells were able to react to factors released by cells with normal-functioning mitochondria^38^, as we also observed in our experiments with ICCM from irradiated Cy143Bwt, which induced a significant increase in the number of γH2AX foci in 143B-Rho0 cells.

In the context of our study, ROS emerge as possible mediators of the observed NTE. Preliminary data with our cell models have shown that 143B-Rho0 cells have, under basal cell culture conditions, low levels of ROS, which do not increase after direct irradiation (Fig. S1B). These data are in accordance with previous reports where it was shown that 143B-Rho0 cells have less amount of ROS^45^. Also, 143B-Rho0 cells do not increase the amount of ROS levels after IR, while Cy143Bwt and Cy143Bmut increase the production of ROS as consequence of direct irradiation (Fig. S1B). Gonçalves *et al*. have previously shown that in addition to the production of low levels of ROS, 143B-Rho0 cells have limited capacity to increase ROS levels when subjected to the mitochondrial complex I inhibitor rotenone^45^. Thereby, it is conceivable to hypothesize that the lack of bystander effects induced by 143B-Rho0 ICCM could be due to their inefficiency to release ROS into the extracellular medium as a consequence of irradiation, in contrast with what happens with Cy143Bwt and Cy143Bmut. However, we should also consider that mtDNA by itself may be a mediator of the NTE induced by Cy143Bwt and Cy143Bmut; contrarily, this would not occur with 143B-Rho0 as they do not have mtDNA. A recent report by Ariyoshi K *et al*. showed that irradiated cells can release mtDNA through exosome-like vesicles to the extracellular media which mediate NTE effects^9^. In fact, in our data we observe the induction of bystander effects by the cybrid cell lines that possess mtDNA (both Cy143Bwt and Cy143Bmut), while 143B-Rho0 cells do not induce these effects.

In this study we verified differential sensitivity to radiation according to mitochondrial fitness, suggesting that modulation of mitochondrial function can have a role in designing therapeutic approaches for radiation resistant cancers.

## Conclusion

In our study, we used cybrid cell models to better clarify the role of mitochondria in the non-targeted effects of radiation exposure. We observed that absence of mtDNA prevented irradiated cells from releasing communicating factors to the culture media, capable of inducing a response in non-irradiated cells. Yet, these cells were still able to respond to factors present in the media from irradiated cells lines. The mtDNA mutation A3243T in tRNA^Leu(UUR)^ gene seemed to slightly modify the intrinsic response to ICCM and its ICCM seemed to induce a different response when compared to ICCM from the wild type cell line. Overall, our results suggest that mitochondrial dysfunction seems to affect the response to IR and could change the response of cells to bystander signals.

The use of cybrid cell lines may be important to clarify the impact of mitochondrial dysfunction on the bystander effects of IR. Establishment and use of cybrid cells, comprising different mtDNA mutations, might be helpful in elucidating the consequences of mitochondrial dysfunction at different levels, in the context of IR. This could have interesting applications for precision medicine in Radiotherapy but also aid in the development of strategies of radiation protection for accidental exposures or space related health effects.

## Supplementary information

Supplementary Information.

## References

[1] Lyng FM, Seymour CB, Mothersill C (2002). Initiation of apoptosis in cells exposed to medium from the progeny of irradiated cells: a possible mechanism for bystander-induced genomic instability?. Radiat. Res..

[2] Sokolov MV (2005). Ionizing radiation induces DNA double-strand breaks in bystander primary human fibroblasts. Oncogene.

[3] Rzeszowska-Wolny J (2009). X-irradiation and bystander effects induce similar changes of transcript profiles in most functional pathways in human melanoma cells. DNA. Repair (Amst)..

[4] Prise KM, O’Sullivan JM (2009). *Radiation-induced bystander signalling in cancer therapy*. Nat. Rev. Cancer.

[5] de Toledo SM (2017). Genomic instability induced in distant progeny of bystander cells depends on the connexins expressed in the irradiated cells. Int. J. Radiat Biol..

[6] Nikitaki Z (2016). Systemic mechanisms and effects of ionizing radiation: A new ‘old’ paradigm of how the bystanders and distant can become the players. Semin Cancer Biol.

[7] Chen S (2009). Up-regulation of ROS by mitochondria-dependent bystander signaling contributes to genotoxicity of bystander effects. Mutat Res.

[8] Zasukhina VFM (2017). *The miRNA as human cell gene activity regulator after ionizing radiation*. Russian Journal of Genetics.

[9] Yoshida KA (2019). Radiation-Induced Bystander Effect is Mediated by Mitochondrial DNA in Exosome-Like Vesicles. Scientific Reports.

[10] Nagasawa H, Little JB (1992). Induction of sister chromatid exchanges by extremely low doses of alpha-particles. Cancer Res..

[11] Mothersill C, Seymour C (1997). Medium from irradiated human epithelial cells but not human fibroblasts reduces the clonogenic survival of unirradiated cells. Int. J. Radiat Biol..

[12] Mukherjee D (2014). *Responses to ionizing radiation mediated by inflammatory mechanisms*. The Journal of Pathology.

[13] Leach JK (2002). Activation of constitutive nitric-oxide synthase activity is an early signaling event induced by ionizing radiation. J. Biol. Chem..

[14] Shao C (2003). Nitric oxide-mediated signaling in the bystander response of individually targeted glioma cells. Cancer Res..

[15] Schreck R, Albermann K, Baeuerle PA (2009). *Nuclear Factor Kb: An Oxidative Stress-Responsive Transcription Factor of Eukaryotic Cells (A Review)*. Free Radical Research Communications.

[16] Zhou H (2008). Mitochondrial function and nuclear factor-kappaB-mediated signaling in radiation-induced bystander effects. Cancer Res..

[17] Finkel T (2011). *Signal transduction by reactive oxygen species*. J. Cell Biol..

[18] Ermakov, A. V. *et al*., Oxidized extracellular DNA as a stress signal in human cells, in Oxidative Medicine and Cellular Longevity. Hindawi. 649747 (2013). 10.1155/2013/649747 PMC3606786 23533696

[19] Mittra I (2017). Cell-free chromatin from dying cancer cells integrate into genomes of bystander healthy cells to induce DNA damage and inflammation. Cell Death Discov.

[20] Tsukimoto M (2015). Purinergic Signaling Is a Novel Mechanism of the Cellular Response to Ionizing Radiation. Biol. Pharm. Bull..

[21] Xu S (2014). MiR-21 is involved in radiation-induced bystander effects. RNA. Biol..

[22] Al-Mayah A (2015). The non-targeted effects of radiation are perpetuated by exosomes. Mutat Res.

[23] Jelonek K, Widlak P, Pietrowska M (2016). The Influence of Ionizing Radiation on Exosome Composition, Secretion and Intercellular Communication. Protein Pept. Lett..

[24] Le M (2017). Exosomes are released by bystander cells exposed to radiation-induced biophoton signals: Reconciling the mechanisms mediating the bystander effect. PLoS One.

[25] Srinivasan TM (2017). *Biophotons as Subtle Energy Carriers*. Int. J. Yoga.

[26] Mothersill C (2020). *Biophotons in radiobiology: inhibitors, communicators and reactors*. Radiation Protection Dosimetry.

[27] Frayn KN (2003). *The glucose-fatty acid cycle: a physiological perspective*. Biochem. Soc. Trans..

[28] Saris NE, Carafoli E (2005). A historical review of cellular calcium handling, with emphasis on mitochondria. Biochemistry (Mosc)..

[29] Kroemer G (2003). *Mitochondrial control of apoptosis: an introduction*. Biochem. Biophys. Res. Commun..

[30] Le M (2018). Modulation of oxidative phosphorylation (OXPHOS) by radiation- induced biophotons. Environ. Res..

[31] Wilkins HM, Carl SM, Swerdlow RH (2014). Cytoplasmic hybrid (cybrid) cell lines as a practical model for mitochondriopathies. Redox Biol.

[32] van Gisbergen MW (2015). How do changes in the mtDNA and mitochondrial dysfunction influence cancer and cancer therapy? Challenges, opportunities and models. Mutat. Res. Rev. Mutat. Res..

[33] Sazonova MA (2018). *Cybrid Models of Pathological Cell Processes in Different Diseases*. Oxid Med Cell Longev.

[34] Nunes JB (2015). OXPHOS dysfunction regulates integrin-beta1 modifications and enhances cell motility and migration. Hum Mol Genet.

[35] Saffran HA (2007). *Herpes simplex virus eliminates host mitochondrial DNA*. EMBO Rep..

[36] Hu Y, Zhu TC (2011). *Backscatter correction factor for megavoltage photon beam*. Med. Phys..

[37] Barbour, J.A. and N. Turner, Mitochondrial stress signaling promotes cellular adaptations, in International Journal of Cell Biology. 2014, Hindawi. 156020. 10.1155/2014/156020 PMC3920668 24587804

[38] Tartier L (2007). Cytoplasmic irradiation induces mitochondrial-dependent 53BP1 protein relocalization in irradiated and bystander cells. Cancer Res..

[39] King MP, Attardi G (1989). Human cells lacking mtDNA: repopulation with exogenous mitochondria by complementation. Science.

[40] Yoshida K (2000). *Role of mitochondrial DNA in radiation exposure*. Radiat Med..

[41] Kulkarni R (2010). Mitochondrial gene expression changes in normal and mitochondrial mutant cells after exposure to ionizing radiation. Radiat Res..

[42] Cloos CR (2009). Mitochondrial DNA depletion induces radioresistance by suppressing G2 checkpoint activation in human pancreatic cancer cells. Radiat Res..

[43] van Gisbergen MW (2017). Distinct radiation responses after *in vitro* mtDNA depletion are potentially related to oxidative stress. PLoS One.

[44] Qin L (2015). CDK1 Enhances Mitochondrial Bioenergetics for Radiation-Induced DNA Repair. Cell. Rep..

[45] Goncalves AP (2011). Involvement of p53 in cell death following cell cycle arrest and mitotic catastrophe induced by rotenone. Biochim. Biophys. Acta..

[46] Tsukimoto, S.K., *et al*., Role of ATP as a Key Signaling Molecule Mediating Radiation-Induced Biological Effects. 10.1177/1559325817690638, 2017. 10.1177/1559325817690638 PMC5318813 28250717

[47] Kostyuk SV (2012). Role of extracellular DNA oxidative modification in radiation induced bystander effects in human endotheliocytes. Mutat. Res..

[48] Kirolikar S (2018). *Prevention of radiation-induced bystander effects by agents that inactivate cell-free chromatin released from irradiated dying cells*. Cell Death & Disease.

