# Supplementary material for: Evaluation of the role of mitochondria in the non-targeted effects of ionizing radiation using cybrid cellular models
Source: Sci Rep. 2020 Apr 9;10:6131. doi: 10.1038/s41598-020-63011-w (PMC7145863; doi:10.1038/s41598-020-63011-w)
Supplement: Supplementary file 1 — Supplementary Information. [file 41598_2020_63011_MOESM1_ESM.pdf]

A

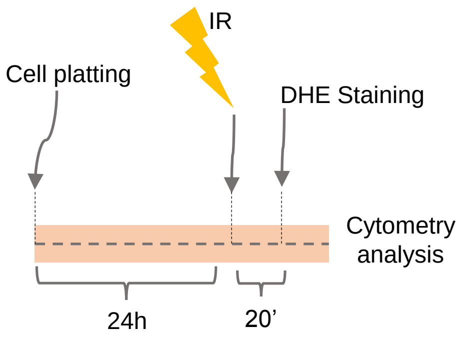

B

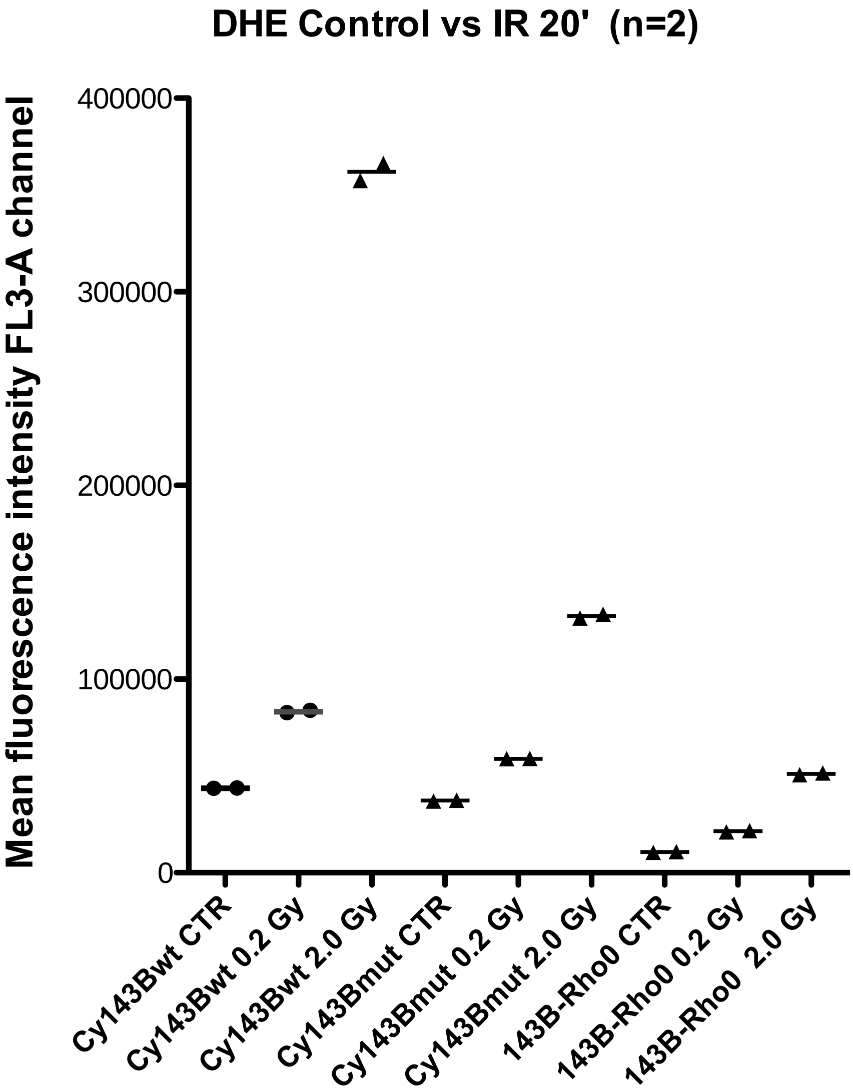

Figure S1 – Irradiation protocol and assesment of ROS levels.

A – Schematic representation of the cell plating and irradiation timeframe used.

B – Evaluation of the levels of superoxide in cells stained with Dihydroethidium (DHE) from Sigma-Aldrich dye and fluorescence analysed in the FL3-A channel on BD Accuri C6.
